# Recent advances in understanding the auditory cortex

**DOI:** 10.12688/f1000research.15580.1

**Published:** 2018-09-26

**Authors:** Andrew J. King, Sundeep Teki, Ben D.B. Willmore

**Affiliations:** 1 Department of Physiology, Anatomy & Genetics, University of Oxford, Oxford, OX1 3PT, UK

**Keywords:** auditory cortex, receptive field, model, map, cognition, plasticity

## Abstract

Our ability to make sense of the auditory world results from neural processing that begins in the ear, goes through multiple subcortical areas, and continues in the cortex. The specific contribution of the auditory cortex to this chain of processing is far from understood. Although many of the properties of neurons in the auditory cortex resemble those of subcortical neurons, they show somewhat more complex selectivity for sound features, which is likely to be important for the analysis of natural sounds, such as speech, in real-life listening conditions. Furthermore, recent work has shown that auditory cortical processing is highly context-dependent, integrates auditory inputs with other sensory and motor signals, depends on experience, and is shaped by cognitive demands, such as attention. Thus, in addition to being the locus for more complex sound selectivity, the auditory cortex is increasingly understood to be an integral part of the network of brain regions responsible for prediction, auditory perceptual decision-making, and learning. In this review, we focus on three key areas that are contributing to this understanding: the sound features that are preferentially represented by cortical neurons, the spatial organization of those preferences, and the cognitive roles of the auditory cortex.

## Introduction

Our seemingly effortless ability to localize, distinguish, and recognize a vast array of natural sounds, including speech and music, results from the neural processing that begins in the inner ear and continues through a complex sequence of subcortical and cortical brain areas. Many aspects of hearing—such as computation of the cues that enable sound sources to be localized or their pitch to be extracted—rely on the processing that takes place in the brainstem and other subcortical structures. It is widely thought to be the case, however, that the auditory cortex plays a critical role in the perception of complex sounds. Although this partly reflects the emergence of response properties, such as sensitivity to combinations of sound features, it is striking how similar many of the properties of neurons in the primary auditory cortex (A1) are to those of subcortical neurons ^1^. Equally important is the growing realization that auditory cortical processing, in particular, is highly context-dependent and integrates auditory (and other sensory) inputs with information about an individual’s current internal state, including their arousal level, focus of attention, and motor planning, as well as their past experience ^2^. The auditory cortex is therefore an integral part of the network of brain regions responsible for generating meaning from sounds, auditory perceptual decision-making, and learning.

In this review, we focus on three key areas of auditory cortical processing where there has been progress in the last few years. First, we consider what sound features A1 neurons represent, highlighting recent attempts to improve receptive field models that can predict the responses of neurons to natural sounds. Second, we examine the distribution of those stimulus preferences within A1 and across the hierarchy of auditory cortical areas, focusing on the extent to which this conforms to canonical principles of columnar organization and functional specialization that are the hallmark of visual and somatosensory processing. Finally, we look at the cognitive role of auditory cortex, highlighting its involvement in prediction, learning, and decision making.

## Spectrotemporal receptive fields

The tuning properties of sensory neurons are defined by their receptive fields, which describe the stimulus features to which they are most responsive. Reflecting the spectral analysis that begins in the inner ear, auditory neurons are most commonly characterized by their sensitivity to sound frequency. The spectrotemporal receptive field ^3, 4^ (STRF) is the dominant computational tool for characterizing the responses of auditory neurons. Most widely used as part of a linear-nonlinear (LN) model, an STRF comprises a set of coefficients that describe how the response of the neuron at each moment in time can be modelled as a linear weighted sum of the recent history of the stimulus power in different spectral channels. Owing to their simplicity, STRFs can be reliably estimated by using randomly chosen structured stimuli, such as ripples ^5– 7^, and relatively small amounts of data. They can then be used, in combination with a static output nonlinearity, to describe and predict neural responses to arbitrary sounds ^8^, making them invaluable tools in understanding the computational roles of single neurons and whole neuronal populations. However, STRFs do not accurately capture the full complexity of the behavior of auditory neurons; for example, multiple studies have shown that there are differences between STRFs estimated by using standard synthetic stimuli and those estimated by using natural sounds ^9– 11^, suggesting that these models systematically fail to fully describe neural responses to complex stimuli.

### Multiple stimulus dimensions

Standard STRF models contain a single receptive field—one set of spectrotemporal weighting coefficients that describes a single time-varying spectral pattern to which the neuron is sensitive. There is no reason, however, to believe that the spectrotemporal selectivity of auditory neurons is so simple. Artificial neural networks are built on the principle that arbitrarily complex computations can be constructed from simple neuron-like computing elements, and cortical neurons are likely to have similarly complex selectivity based on nonlinear combination of the responses of afferent neurons with simpler selectivity. Capturing this complexity requires STRF models that nonlinearly combine the responses of neurons to multiple stimulus dimensions.

With a maximally informative dimensions approach, it has been found that multiple stimulus dimensions are required to describe the responses of neurons in A1 ^12, 13^, whereas a similar approach requires only a single dimension to describe most neurons in the inferior colliculus (IC) in the midbrain ^14^. This suggests that neuronal complexity is higher in the cortex and that this complexity can be captured by nonlinear combination of the responses of multiple simpler units, a finding that also applies to high-level neurons in songbirds ^15, 16^. Two recent articles have successfully shown that neural networks are an effective way to model these interactions. Harper *et al*. ^17^ used a two-layer perceptron to describe the behavior of neurons in ferret auditory cortex, and Kozlov and Gentner ^16^ used a similar network to describe high-level auditory neurons in the starling. To achieve this, both studies used neural networks of intermediate complexity, thus avoiding an explosion in the number of parameters that must be fitted. They also used careful regularization—where the model is optimized subject to a penalty on parameter values. Regularization effectively reduces the number of parameters that must be fitted, meaning that models can be fitted accurately using smaller datasets ^18^. This approach enabled both studies to show that auditory neurons can be better described by a model which takes into account the tuning of multiple afferent neurons (hidden units), suggesting that the selectivity of individual neurons is the result of a combination of information about multiple stimulus dimensions.

Other studies using synthetic stimuli also indicate that cortical neurons integrate different sound features, including frequency, spectral bandwidth, level, amplitude modulation over time, and spatial location ^19, 20^. By using an online optimization procedure (see earlier work by deCharms *et al*. ^21^) to dynamically generate sounds that varied along these dimensions, the authors of these studies were able to efficiently search stimulus space and constrain their model within a few minutes of stimulus presentation. They also found that individual neurons multiplex information about multiple stimulus dimensions and that neuronal responses to multidimensional stimuli could not be predicted simply from responses to low-dimensional stimuli.

Taken together, these studies suggest that the successor of the STRF will be a model that allows multiple STRF-like elements to be combined in nonlinear ways. The development of such models will require large datasets that include neuronal responses to complex, natural stimuli.

### Dynamic changes in tuning

Static multidimensional models capture complex selectivity for the recent spectral structure of sounds. However, several studies have highlighted the importance of dynamic changes in the response properties of auditory cortical neurons ^22– 26^. Most real-life soundscapes are characterized by constant changes in the statistics of the sounds that reach the ears. Neurons rapidly adapt to stimulus statistics—for example, stimulus probability, contrast, and correlation structure—and adjust their sensitivity in response to behavioral requirements ^27– 29^. This can produce representations that are invariant to some changes in sound features and robustly selective for other features ^30^.

Several authors have incorporated such adaptation by adding a nonlinear input stage to the standard LN model ^25, 31– 33^. Willmore *et al*. ^33^ explicitly incorporated the behavior of afferent neurons into a model of A1 spectrotemporal tuning. In both the auditory nerve ^34^ and IC ^35^, dynamic coding occurs in the form of adaptation to mean sound level. When the mean sound level is high, neurons shift their dynamic ranges upwards, so that neuronal responses are relatively invariant to changes in background level. Because these structures are precursors of the auditory cortex, it makes sense to incorporate IC adaptation into the input stage of a model of auditory cortex and Willmore *et al*. ^33^ found that this improved the performance of their model of cortical neurons.

Neurons in the A1 of the ferret show compensatory adaptation to sound contrast (that is, the variance of the sound level distribution) ^24, 36^. When the contrast of the input to a given neuron is high, the gain of the neuron is reduced, thereby making it relatively insensitive to changes in sound level. When the contrast of the input is low, the gain of the neuron rises, increasing its sensitivity. This adaptive coding therefore tends to compensate for changes in sound contrast. Adaptation to the mean and variance of sound level is specifically beneficial for the representation of dynamic sounds against a background of constant noise. If the noise is statistically stationary (that is, the mean and variance are fixed), then the effect of adaptation is to minimize the responses of cortical neurons to the background. Thus, the neuronal responses depend mainly on the dynamic foreground sound and are relatively invariant to its contrast. This enables cortical neurons to represent complex sounds using a code that is relatively robust to the presence of background noise. Similar noise robustness has been shown independently in the songbird ^37^ and in decoding studies applied to populations of mammalian cortical neural responses ^30, 38^.

### Normative models

To fully understand how sounds are represented by neurons in the auditory cortex, we need to ask why this particular representation has been selected, whether by evolution or by developmental processes. One approach to addressing this question is to build normative computational models. Normative models embody certain constraints that are hypothesized to be important for determining the neural representation. By building normative models and comparing their representations with real physiological representations, it is possible to test hypotheses about which constraints determine the structure of the neural codes used in the brain. For example, Olshausen and Field ^39^ built a neural network and trained it to produce a sparse, generative representation of natural visual scenes. Once trained, the network’s units exhibited receptive fields with many similarities to those found in neurons in the primary visual cortex (V1), suggesting that V1 itself may be optimized to produce sparse representations of natural visual scenes.

**Figure 1.**
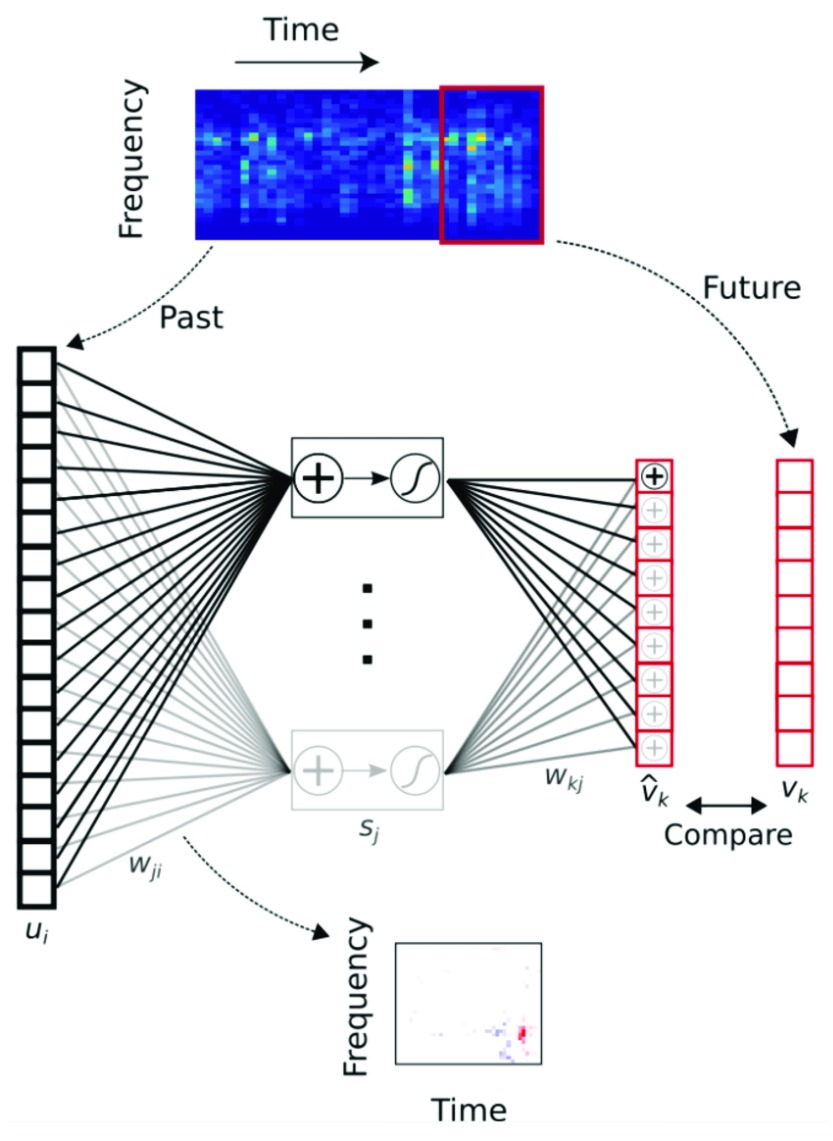
Neuronal selectivity in the auditory cortex is optimized to represent sound features in the recent sensory past that best predict immediate future inputs. A feedforward artificial neural network was trained to predict the immediate future of natural sounds (represented as cochleagrams describing the spectral content over time) from their recent past. This temporal prediction model developed spectrotemporal receptive fields that closely matched those of real auditory cortical neurons. A similar correspondence was found between the receptive fields produced when the model was trained to predict the next few video frames in clips of natural scenes and the receptive field properties of neurons in primary visual cortex. Model nomenclature: *s _j_*, hidden unit output; *u _i_*, input—the past; *v _k_*, target output—the true future; ${\hat{v}}_{k}$, output—the predicted future; *w _ji_*, input weights (analogous to cortical receptive fields); *w _kj_*, output weights. Reprinted from Singer *et al*. ^42^.

Carlson *et al*. ^40^ trained a neural network so that it produced a sparse, generative representation of natural auditory stimuli. They found that the model learned STRFs whose spectral structure resembled that of real neurons in the auditory system, suggesting that the auditory system may be optimized for sparse representation. Carlin and Elhilali ^41^ showed that optimizing model neural responses to produce a code based on sustained firing rates also revealed feature preferences similar to those of auditory neurons. Recently, Singer *et al*. ^42^ observed that the temporal structure of the STRFs in sparse coding models (which generally have envelopes that are symmetrical in time) is very different from those of real neurons in both V1 and A1, where neurons are typically most selective for recent stimulation and have envelopes that decay into the past. These authors trained networks to predict the immediate future of either natural visual or auditory stimuli and found that the networks developed receptive fields that closely matched those of real visual and auditory cortical neurons, including their asymmetric temporal envelopes ( Figure 1). This suggests that neurons in these areas may be optimized to predict the immediate future of sensory stimulation. Such a representation may be advantageous for acting efficiently in the world using neurons, which introduce inevitable delays in information transmission.

As discussed above, STRFs do not provide a complete description of the stimulus preferences of auditory neurons. It is therefore important to extend the normative approach into more complex models in order to generate hypotheses about what nonlinear combinations of features we might expect to find in the auditory system. To address this question, Młynarski and McDermott ^43^ trained a hierarchical, generative neural network to represent natural sounds and found that some units in the second layer of the model developed sensitivity to combinations of elementary sound features but that others developed opponency between sound features. By providing insights into how different parameters interact to determine their response properties, these modelling approaches can generate testable hypotheses about the possible roles played by auditory neurons at different levels of the processing hierarchy.

## Origin of auditory cortical tuning properties

The receptive field properties of auditory cortical neurons are derived from their ascending thalamic inputs and are shaped by recurrent synaptic interactions between excitatory neurons as well as by local inhibitory inputs. The importance of ascending inputs in determining the functional organization of the auditory pathway is illustrated by the presence of tonotopic maps at each processing level, which have their origin in the decomposition of sounds into their individual frequencies along the length of the cochlea in the inner ear. The presence of tonotopic maps in the cortex can be demonstrated by using a variety of methods, including the use of functional brain imaging in humans ^44^. However, this is a relatively coarse approach and the much finer spatial resolution provided by *in vivo* two-photon calcium imaging suggests that the spatial organization varies across the layers of A1 and that frequency tuning in the main thalamorecipient layer 4 is more homogenous than in the upper cortical layers ^45^. Whilst this laminar transformation is consistent with a possible integration of inputs in the superficial layers, which might contribute to the emergence of sensitivity to multiple sound features, it turns out that the thalamic input map to A1 is surprisingly imprecise ^46^. The significance of this for the generation of cortical response properties remains to be investigated, but heterogeneity in the frequency selectivity of ascending inputs might provide a substrate for the contextual modulation of cortical response properties.

In the last few years, progress has been made in determining how the local circuitry of excitatory and inhibitory neurons contributes to auditory cortical response properties ^47– 50^. For example, both parvalbumin-expressing and somatostatin-expressing inhibitory interneurons in layers 2/3 have been implicated in regulating the frequency selectivity of A1 neurons ^48, 50^, while manipulating the activity of parvalbumin-expressing neurons, the most common type of cortical interneuron, has been shown to alter the behavioral performance of mice on tasks that rely on their ability to discriminate different sound frequencies ^49^. Furthermore, local inhibitory interneurons are involved in mediating the way A1 responses change according to the recent history of stimulation ^51– 54^, and it is likely that dynamic interactions between different cell types are responsible for much of the context-dependent modulation that characterizes the way sounds are processed in the auditory cortex. Thus, in addition to being present at subcortical levels, adaptive coding of auditory information can arise *de novo* from local circuit interactions in the cortex.

## Beyond tonotopy

A tonotopic representation is defined by a systematic variation in the frequency selectivity of the neurons from low to high values. A long-standing and controversial question regarding the functional organization of A1 and other brain areas that contain frequency maps is how neuronal sensitivity to other stimulus features is represented across the isofrequency axes. In the visual cortex of carnivores and primates, neuronal preferences for different stimulus features—orientation, spatial frequency and eye of stimulation—are overlaid so that they are all represented at each location within a two-dimensional map of the visual field ^55^. In a similar vein, functional magnetic resonance imaging (fMRI) studies in humans ^56^ and macaque monkeys ^57^ have revealed that core auditory fields, including A1, contain a gradient in sensitivity to the rate of amplitude modulation, which is arranged orthogonally to the tonotopic map. In other words, a temporal map seems to exist within the cortical representation of each sound frequency.

By contrast, analysis of the activity of large samples of neurons within the upper layers of mouse auditory cortex has failed to provide evidence for such topography. Instead, neurons displaying similar frequency tuning bandwidth ^58^, preferred sound level ^58^, and sensitivity to changes in sound level ^59^ or to differences in level between the ears ^60^—an important cue to localizing sound sources—form intermingled clusters across the auditory cortex. Within the isofrequency domain, clearer evidence for segregated processing modules with distinct spectral integration properties or preferences for other sound features has been obtained in monkeys ^61^ and cats ^62^. It therefore remains possible that the more diffuse spatial arrangement observed in mice may be a general property of sensory cortex in this species ^63^ or more generally of animals with relatively small brains. Although multiple, interleaved processing modules for behaviorally relevant sound features may exist to varying degrees in different species, our understanding of how they map onto the tonotopic organization of A1 or other auditory areas is far from complete.

Vertical clustering of neurons with similar tuning properties also represents a potentially important aspect of the functional organization of the cerebral cortex. Compared with other sensory systems, there has been less focus on the columnar organization of auditory cortex. As previously mentioned, there is evidence for layer-specific differences in the frequency representation in auditory cortex ^45^ and this has been shown to extend to other response properties ^64, 65^. At the same time, ensemble activity within putative cortical columns may play a role in representing auditory information ^66, 67^.

## Representing sound features in multiple cortical areas

As with other sensory modalities, the auditory cortex is subdivided into a number of separate areas, which can be distinguished on the basis of their connections, response properties, and (to some extent) the auditory perceptual deficits that result from localized damage. That these areas are organized hierarchically is clearly illustrated by neuroimaging evidence in humans for the involvement of sequential cortical regions in transforming the spectral features of speech into its semantic content ^68, 69^. Furthermore, by training a neural network model on speech and music tasks, Kell *et al*. ^70^ found that the best performing model architecture separated speech and music into separate pathways, in keeping with fMRI responses in human non-A1 ^71^. Neuroimaging evidence in monkeys also suggests that analysis of auditory motion may involve different pathways from those engaged during the processing of static spatial information ^72^. In both human and non-human primates, the concept of distinct ventral and dorsal processing streams is widely accepted. These were initially assigned to “what” and “where” functions, respectively, but their precise functions, and the extent to which they interact, continue to be debated ^73– 76^.

Evidence for a division of labor among the multiple areas that comprise the auditory cortex extends to other species, as illustrated, for example, by the finding that anatomically segregated regions of mouse auditory cortex can be distinguished by differences in the frequency selectivity of the neurons and in their sensitivity to frequency-modulated sweeps ^77^. Nevertheless, the question of whether a particular aspect of auditory perception is localized to one or more of those areas is, in some ways, ill posed. Given the extensive subcortical processing that takes place and the growing evidence for multidimensional receptive field properties in early cortical areas ^19, 20, 78^, it is likely that neurons convey information about multiple sound attributes. Indeed, recent neuroimaging studies have demonstrated widespread areas of activation in response to variations in spatial ^79^ or non-spatial ^80^ parameters, adding to earlier electrophysiological recordings which indicated that sensitivity to pitch, timbre, and location is distributed across several cortical areas ^81^.

## Decoding auditory cortical activity

Even where different sound features are apparently encoded by the same cortical regions, differences in the evoked activity patterns may enable them to be distinguished. For example, auditory cortical neurons can unambiguously represent more than one stimulus parameter by independently modulating their spike rates within distinct time windows ^78^. Furthermore, a recent fMRI study demonstrated that although variations in pitch or timbre—two key aspects of sound identity—activate largely overlapping cortical areas, they can be distinguished by using multivoxel pattern analysis ^80^. Studies like these hint at how the brain might solve the problem of perceptual invariance—recognizing, for example, the melody of a familiar tune even when it is played on different musical instruments irrespective of where they are located.

This type of approach is part of a growing trend to investigate the decoding or reconstruction of sound features from the measured responses of populations of neurons (or other multidimensional measures of brain activity, such as fMRI voxel responses ^82^ or electroencephalography signals). Indeed, Yildiz *et al*. ^83^ found that a normative decoding model of auditory cortex described neuronal response dynamics better than some classic encoding models that define the relationship between the stimulus and the neural response. It is therefore possible that the responses of populations of cortical neurons can be more accurately understood in terms of decoding rather than encoding.

The application of decoding techniques has provided valuable insights into how neural representations change under different sensory conditions, such as in the presence of background noise ^30, 38, 84, 85^, or when specific stimuli are selectively attended ^86– 89^. They can also help to identify the size of the neural populations within the cortex from which auditory information needs to be read out in order to account for behavior ^90– 92^ and the way in which stimulus features are represented there. For example, evidence from electrophysiological ^93, 94^, two-photon calcium imaging ^60^ and fMRI ^95, 96^ studies is all consistent with an opponent-channel model in which the location of sounds in the horizontal plane, at least based on interaural level differences, is decoded from the relative activity of contralaterally and ipsilaterally tuned neurons within each hemisphere. Furthermore, based on the accuracy with which spectrotemporal modulations in a range of natural sounds could be reconstructed from high-resolution fMRI signals, Santoro *et al*. ^97^ concluded that even early stages of the human auditory cortex may be optimized for processing speech and voices.

## Auditory scene analysis

Separating a sound source of interest from a dynamic mixture of potentially competing sounds is a fundamental function of the auditory system. In order to perceptually isolate a sound source as a distinct auditory object ^98^, its constituent acoustic features like pitch, timbre, intensity, and location need to be individually analyzed and then grouped together to form a coherent perceptual representation. Although neural computations underlying scene analysis have been demonstrated at different subcortical levels ^99– 101^, the cortex is the hub where stimulus-driven feature segregation and top-down attentional selection mechanisms converge ^102^. Indeed, several recent studies suggest a critical role for the auditory cortex in forming stable perceptual representations based on grouping and segregation of spectral, spatial, and temporal regularities in the acoustic environment ^103– 115^.

Whilst cortical spike-based accounts of auditory segregation of narrowband signals rely on mechanisms of tonotopicity, adaptation, and forward suppression ^116^, recent work has highlighted the importance of temporal coherence ^113, 117– 119^ and oscillatory sampling ^120– 122^ in driving dynamic segregation of attended broadband stimuli. Results from human electro-corticography experiments suggest that low-level auditory cortex encodes spectrotemporal features of selectively attended speech ^86, 87^. Speech representations derived from high-gamma (75- to 150-Hz) local field potential activity in non-A1 of subjects listening to two speakers talking simultaneously are dominated by the spectrotemporal features of whichever speaker attention is drawn to ^86^. Moreover, the attention-modulated responses were found to predict the accuracy with which target words were correctly detected in the two-speaker speech mixture, revealing a neural correlate of “cocktail party” listening ^86^. Attentional modulation of the representation of multiple speech sources increases along the cortical hierarchy ^114^, and a similar finding has been reported for the emergence of task-dependent spectrotemporal tuning in neurons in ferret cortex ^123^.

## Prediction

The predictability of sounds plays an important role in processing natural sound sequences like speech and music ^124^. A prominent generative model of perception is based on the concept of predictive coding; that is, the brain learns to minimize the prediction error between internal predictions of sensory input and the external sensory input ^125, 126^. This model has been successfully applied to explain the encoding of pitch in a hierarchical fashion in distributed areas of the auditory cortex ^127^. Furthermore, distinct oscillatory signatures for the key variables of predictive coding models, including surprise, prediction error, prediction change, and prediction precision, have all been demonstrated in the auditory cortex ^128^. Most of the work in this area at the level of individual neurons has focused on stimulus-specific adaptation, a phenomenon in which particular sounds elicit stronger responses when they are rarely encountered than when they are common ^22^. Recent work has indicated that A1 neurons exhibit deviance or surprise sensitivity ^129^ and encode prediction error ^124^ and that prediction error signals increase along the auditory pathway ^130^. Furthermore, the auditory cortex encodes predictions for not only “what” kind of sensory event is to occur but also “when” it may occur ^131^.

## Behavioral engagement and auditory decision-making

Further evidence for dynamic processing in the auditory cortex has been obtained from studies demonstrating that engagement in auditory tasks modulates both spontaneous ^132, 133^ and sound-evoked ^92, 133– 135^ activity, as well as correlations between the activity of different neurons ^92, 136^, in ways that enhance behavioral performance. Furthermore, changes in task reward structure can alter A1 responses to otherwise identical sounds ^28^, and other studies suggest that the auditory cortex is part of the network of brain regions involved in maintaining perceptual representations during memory-based tasks ^137, 138^. Although the source of these modulatory signals is not yet fully understood, accumulating evidence suggests the involvement of top-down inputs from areas such as the parietal and frontal cortices ^88, 139– 141^ as well as the neuromodulatory systems discussed in the next section. In addition, inputs from motor cortex have been shown to suppress both spontaneous and evoked activity in the auditory cortex during movement ^142, 143^.

The notion of the auditory cortex as a high-level cognitive processor is also supported by investigations into perceptual decision-making. Traditionally, decision making is a function that has been attributed to parietal and frontal cortex in association with subcortical structures like the basal ganglia. However, recent work suggests that the auditory cortex not only encodes physical attributes of task-relevant stimuli but also represents behavioral choice and decision-related signals ^92, 144– 146^. Tsunada *et al*. ^147^ provided direct evidence for a role for auditory cortex in decision making: they found that micro-stimulation of the anterolateral but not the mediolateral belt region of the macaque auditory cortex biased behavioral responses toward the choice associated with the preferred sound frequency of the neurons at the site of stimulation.

## The auditory cortex and learning

An ability to learn and remember features in complex acoustic scenes is crucial for adaptive behavior, and plasticity of sound processing in the auditory cortex is an integral part of the circuitry responsible for these essential functions. Robust learning often occurs without conscious awareness and persists for a long time. Agus *et al*. ^148^ demonstrated that human participants can rapidly learn to detect a particular token of repeated white noise and remember the same pattern for weeks ^149^ in a completely unsupervised fashion. Functional brain imaging experiments have implicated the auditory cortex as well as the hippocampus in encoding memory representations for such complex scenes. The underlying mechanisms of this rapid formation of robust acoustic memories are not clear, but recent psychoacoustical experiments suggest a computation based on encoding summary statistics ^150^.

Recognition of structure in complex sequences based on passive exposure is also imperative for acquiring knowledge of the various rules manifest in speech and language ^151^. Electrophysiological recordings from songbird forebrain areas that correspond to the mammalian auditory cortex have revealed evidence for statistical learning, expressed as a decrease in the spike rate for familiar versus novel sequences ^152^. Following passive learning with sequences of nonsense speech sounds that contained an artificial grammar structure, exposure to violations in this sequence activated homologous cortical areas ^153^ and modulated hierarchically nested low-frequency phase and high-gamma amplitude coupling in the auditory cortex in a very similar way in humans and monkeys ^154^. Interestingly, this form of oscillatory coupling is thought to underlie speech processing in the human auditory cortex ^155^, suggesting that it may represent an evolutionarily conserved strategy for analyzing sound sequences.

Not all learning, however, occurs without supervision, and reinforcement in the form of reward or punishment is also key for successful learning outcomes. As in other sensory modalities, training can produce improvements in auditory detection and discrimination abilities, including linguistic and musical abilities ^156^. A number of studies have reported that auditory perceptual learning is associated with changes in the stimulus-encoding response properties of A1 neurons ^157^. This often entails an expansion in the representation of the stimuli on which subjects are trained, and the extent of the representational plasticity is thought to encode both the behavioral importance of these stimuli and the strength of the associative memory ^158^. Nevertheless, there have been few attempts to show that A1 plasticity is required for auditory perceptual learning. Indeed, training-induced changes in the functional organization of A1 have been found to disappear over time, even though improvements in behavioral performance are retained ^159, 160^. However, a recent study in which gerbils were trained on an amplitude-modulation detection task found both a close correlation between the magnitude and time course of cortical and behavioral plasticity and that inactivation of the auditory cortex reduced learning without affecting detection thresholds ^161^. Cortical inactivation has also been shown to impair training-dependent adaptation to altered spatial cues resulting from plugging of one ear ^162^. This is consistent with physiological evidence that the encoding of different spatial cues in the auditory cortex is experience-dependent, changing in ways that can explain the recovery of localization accuracy following exposure to abnormal inputs ^94, 163, 164^.

The alterations in cortical response properties that accompany perceptual learning are likely driven by top-down inputs that determine which stimulus features to attend to ^165^. Several neuromodulatory systems, including the cholinergic basal forebrain ^166, 167^ and the noradrenergic locus coeruleus ^168, 169^, play an important role in auditory processing and plasticity and appear to provide reinforcement signals and information about behavioral context to auditory cortex. It has recently been shown that cholinergic inputs primarily target inhibitory interneurons in the auditory cortex ^170, 171^, which suggests a possible basis by which the balance of cortical excitation and inhibition is transiently altered during learning ^172^.

Although its involvement in learning is probably one of the most important functions of the auditory cortex, there is growing evidence for a specific role for its outputs to other brain areas. For instance, selective strengthening of auditory corticostriatal synapses has been observed over the course of learning an auditory discrimination task ^173^, while the integrity of A1 neurons that project to the IC is required for adaptation to hearing loss in one ear ^174^. Indeed, it is likely that descending corticofugal pathways play a more general role in auditory learning ^175, 176^ as well as other aspects of auditory perception and behavior ^177– 180^.

## Conclusions

The studies outlined in this article show how neurons in the auditory cortex encode sounds in ways that are directly relevant to behavior. Auditory cortical processing depends not only on the sounds themselves but also on the individual’s internal state, such as the level of arousal, and the sensory and behavioral context in which sounds are detected. Therefore, to understand how cortical neurons process specific sound features, we have to consider the complexity of the auditory scene and the presence of other sensory cues as well as factors such as motor activity, experience, and attention. Indeed, the auditory cortex seems to play a particularly important role in learning and in constructing memory-dependent perceptual representations of the auditory world.

We are now beginning to understand the computations performed by auditory cortical neurons as well as the role of long-range inputs and local cortical circuits, including the participation of specific cell types, in those computations. Research over the last few years has also provided insights into the way information flows within and between different auditory cortical areas as well as the complex interplay between the auditory cortex and other brain areas. In particular, through its extensive network of descending projections, cortical activity can influence almost every subcortical processing stage in the auditory pathway, but we are only beginning to understand how those pathways contribute to auditory function. Further progress in this field will require the application of coordinated computational and experimental approaches, including the increased use of methods for measuring, manipulating, and decoding activity patterns from populations of neurons.
